# Does the Degree of Fatness and Muscularity Determined by Ultrasound Method Affect Sows’ Reproductive Performance?

**DOI:** 10.3390/ani10050794

**Published:** 2020-05-03

**Authors:** Damian Knecht, Sebastian Środoń, Katarzyna Czyż

**Affiliations:** Institute of Animal Breeding, Faculty of Biology and Animal Sciences, Wroclaw University of Environmental and Life Sciences, 50-375 Wroclaw, Poland; damian.knecht@upwr.edu.pl (D.K.); sebastian.srodon@o2.pl (S.Ś.)

**Keywords:** gilts, litter, parity, sows, ultrasonography, Aloka SSD-500

## Abstract

**Simple Summary:**

One of the factors strongly affecting the profitability of animals breeding is their reproductive performance. The measurement of gilts and sows’ fatness and muscularity levels can be a useful tool in forecasting their future reproductive parameters, and thus possible selection with respect to these parameters, which is a significant issue from a practical breeders’ point of view. The aim of the study was an evaluation of the fatness and muscularity of pure-bred and hybrid gilts measured using an Aloka SSD-500 device in three consecutive parities, and the relationship between these features and reproductive performance parameters such as litter traits at birth and weaning, pregnancy length and weaning to service interval. It was observed that fatness degree affected the reproductive parameters of females. The females with higher values of fatness traits achieved better farrowing rate and higher numbers of born piglets, and decreased mortality, higher gains of piglets and higher body weight at weaning, as well as a shortening of the weaning-to-service interval were noted. Intramuscular fat content did not affect reproductive parameters. Muscularity also affected reproductive performance parameters, except gestation period. The lowest reproductive indices were found in females with too-high muscularity levels.

**Abstract:**

The fatness and muscularity of Polish Landrace, Polish Large White gilts and sows and their hybrids were determined on the basis of ultrasound measurements in three consecutive parities, and then the relationship between these parameters and reproductive performance was established. Ultrasound measurements demonstrated the highest fat thickness in first parity and the highest fat area over LD muscle in hybrid gilts (PL × PLW). Pure-bred gilts were characterized by poorer muscularity. Fatness level affected the reproductive parameters of females in which the thickness of backfat in UP2 point was above 22.25 mm, the thickness of backfat in UP4 point was above 17.36 mm and the fat area over LD muscle was above 25.81 cm^2^. These females achieved better farrowing rates and higher numbers of born piglets. Decreased mortality, higher gains of piglets and higher body weight at weaning were observed, and the weaning to service interval was shortened in fatter females. Intramuscular fat content did not affect reproductive parameters. Muscularity negatively affected reproductive performance parameters, except gestation period. Too-high muscularity was related to the lowest levels of reproductive indices. The analysis of gilts and sows’ fatness and muscularity levels can help to predict their reproductive performance in the future and thus optimize production results.

## 1. Introduction

The efficiency of reproduction and the level of productivity of sows determine to a large extent both the profitability of production and the quality of piglets for fattening. Therefore, the variables influencing the reproductive potential of sows should be regularly examined. Intensive selection of pigs to improve their leanness has contributed to a significant increase in this index, not only in Poland, but also in other countries. Analyzing the changes in the slaughter traits of gilts of the maternal Polish Large White (PLW) and Polish Landrace (PL) breeds in recent years, it can be observed that the meat content in the carcass increased to the level of about 60% [1].

In order to maximize sow productivity, it is important to know about the growth potential, slaughter potential and the impact of improvement on reproductive performance. The relationships between the degree of muscularity and fatness of gilts and their further use is not clear, showing both positive and negative effects [2]. An important issue, therefore, seems to be the precise demonstration of the effect of growth parameters on the reproductive use of females.

Many researchers have searched for the current response of reproductive functions to slaughter potential raising. Some emphasize that reproductive traits should be taken into account in the development of breeding strategies aimed at improving fattening and slaughter traits. Many authors confirm the negative impact of selection focused solely on improving fattening and slaughter traits, which, in turn, leads to a deterioration in some reproductive performance parameters, and this process is most clearly evident in gilts [3,4]. The reproductive performance may be adversely affected by both an insufficient and excessive degree of fatness and muscularity [5]. According to Tummaruk et al. [3], low fat reserves in sow’s body has a negative impact on future reproductive performance results, which may cause, e.g., faster culling due to poor animal condition and lactation disturbances.

A higher growth rate can accelerate the first estrus symptoms. It should be emphasized that this is an extremely important issue for the proper management and use of the economic opportunities of the basic stock. It should be noted that, as a result of long-term and unilateral selection for leanness, the level of fat cover of gilts at the time of their first mating or insemination decreased significantly. This is related, inter alia, to the negative leanness–fatness correlation, which means that with the increase in meat content, the gilt’s fatness level is subject to a decrease [6,7].

According to Quinton et al. [8], the most important reproductive indicators from an economic point of view are the number of piglets born alive and the number of piglets at weaning, and the weaning weights at the same time. One of the most important objectives in both the breeding and keeping of pigs is to improve the population in terms of economically significant parameters, which undoubtedly include features related to reproductive performance. The level of these features may be related to the degree of fatness and muscularity at the time of first mating or insemination of gilts [9].

The aim of the study was determination of fatness and muscularity of pure-bred gilts and hybrids on the basis of intravital ultrasound measurements in three consecutive parities, and then to define the relationship between the degree of fatness and muscularity of gilts and sows and their reproductive performance determined on the basis of litter parameters at birth and weaning and the length of the weaning to service interval.

## 2. Materials and Methods

The experiment was performed in the years 2013–2014 in an industrial pig-fattening farm situated in Poland, in the Opolskie Voivodeship on 450 gilts, maintained in identical production conditions. The gilts were divided into three groups depending on the genotype: 150 Polish Landrace (PL) gilts, 150 Polish Large White (PLW) gilts, 150 hybrid gilts (PL × PLW). Gilts at the time of insemination were at similar weight (about 129 kg), age (about 252 days, and had a similar average daily gain from birth to the first insemination (about 513 g). The gilts were used for research purposes from a one to three parity. The gilts and sows were kept in individual pens with an area of 1.20 m^2^ equipped with a slatted floor until 4 weeks after insemination. In the period between 4 weeks after insemination and 1 week before farrowing, the females were group-housed in a pen with 25 females on a partially slatted floor. The area of the pen for gilts was 1.64 m^2^/animal, including 0.95 m^2^ of solid floor. The area of the pen for sows was 2.25 m^2^/animal, and the solid floor area was 1.30 m^2^.

The animals were fed in accordance with the recommendations of the Jan Kielanowski Institute of Animal Physiology and Nutrition—National Research Institute of the Polish Academy of Sciences, according to the Pig Nutrition Standards [10], with constant access to water. The feed dose for the female until 90 days of pregnancy contained 11.8 MJ of metabolic energy, 143.6 g of digestible protein, 6.4 g of lysine, 5.3 g of methionine with cystine, 8.2 g of calcium, 6.3 g of phosphorus and 2.7 g of sodium. The feed dose for gilt/sow over 90 days of pregnancy and lactating gilt/sow was 12.5 MJ of metabolic energy, 170.2 g of digestible protein, 10.1 g of lysine, 6.5 g of methionine with cystine, 9.9 g of calcium, 7.2 g of phosphorus and 3.6 g of sodium. Until the 90th day of pregnancy, each female received about 2.80 kg of feed per day, while after this period their diet was increased to 3.30 kg.

The animals were maintained in conditions meeting the requirements of the Ordinance of the Minister of Agriculture and Rural Development of 28 June 2010 on the minimum conditions for maintaining farm animal species [11]. The study was carried out in accordance with the laws and regulations of Poland, Act of 15 January 2015, on the protection of animals used for scientific and educational purposes [12].

Females in estrus were identified twice a day with the help of a search boar (visual and olfactory contact). The estrus was diagnosed by a standing reflex after a back pressure test. The detection of estrus started from the 170th day of gilts’ life. The gilts were inseminated in the third estrus. Both gilts and sows were inseminated twice: the first time 12 h after the onset of estrus symptoms, and the second time 12 to 18 h after the first insemination. All females were inseminated with Duroc × Pietrain (D × P) boar semen. Litter equalization was performed within the genetic lines.

Ultrasound evaluation of fatness and muscularity was performed during insemination procedures using the Aloka SSD-500 device equipped with a 17 cm UST-5044 head with a frequency of 3.5 MHz. The use of the apparatus in combination with dedicated computer software enabled the measurements described in Table 1. Linear and surface measurements were performed according to the methodology provided by Tyra et al. [13], while intramuscular fat measurement was performed using the method provided by Schulte et al. [14].

The next stage of the study was an evaluation of the reproductive performance of gilts, in which the degree of fatness and muscularity were determined using the Aloka SSD-500 apparatus. The following reproductive indices were analyzed: farrowing rate based on two insemination procedures outcomes (%), number of piglets born in total (head), number of alive-born piglets (head), number of dead-born piglets (including stillborn ones, dead during parturition and mummified fetuses) (head), number of weaned piglets (head), mortality of piglets until weaning day (head), total litter weight at 1st day (kg), average piglet weight at 1st day (kg), total litter weight at weaning (kg), average piglet weight at weaning (kg), average body weight gains of piglets from birth to weaning (g), gestation period (days), weaning to service interval (days).

For each ultrasound measurement, after taking into account the distribution of its variables in the population, two levels of the examined factor were determined on the basis of the mean and standard deviation, in such a way that there is an equal number of individuals within each subgroup, within each breed. Next, it was analyzed whether the level of fatness and muscularity at the moment of insemination can influence the reproductive performance results in terms of the examined indices. It was analyzed whether there are statistical differences between the female genotypes with respect to different degrees of fatness and muscularity.

In order to check the repeatability of the results, each ultrasound measurement was performed three times, then the arithmetic mean of the three measurements was calculated. The data were checked for normality of distribution using Kolmogorov–Smirnov test (K-S) with Lilliefors correction. In addition, Brown–Forsythe test (B-F) was used to determine whether the distributions of variables have the same variance. The numerical material was compiled statistically using STATISTICA ver. 10 software (StatSoft Poland Ltd., Krakow, Poland). Verification of the data concerning the degree of fatness and muscularity determined during insemination was performed on the basis of two-factor analysis of variance. The research model was as follows:

x_ijk_ = µ + α_i_ + β_j_ + (αβ)_ij_ + ε_ijkl;_
x_ijk_—value of the dependent variable (fatness and muscularity degree);
µ—overall average;
α_i_—main effect of the i-th breed;
β_j_—main effect of j-th insemination order;
(αβ)_ij_—effect of i-th breed interaction with j-th insemination order;
εijk—random error with a normal distribution with an average of zero and a variance σ^2^.

Reproductive performance was studied using the three-factor analysis of variance (ANOVA). The first factor was the breed of females participating in the experiment. The second factor was the parity (except for the parameter of farrowing rate, where the order of insemination was taken into account). The third factor was the degree of fatness and muscularity. In the model, the degree of fatness was used interchangeably with the degree of muscularity. The following mixed model was used:

x_ijk_ = µ + α_i_ + β_j_ + γ_k_ + (αβ)_ij_ + (αγ)_ik_ + (βγ)_jk_ + (αβγ)_ijk_ + ε_ijkl;_
x_ijk_—value of the dependent variable (reproductive parameter);
µ—overall average;
α_i_—main effect of the i-th breed;
β_j_—main effect of the j-th parity or insemination order;
γ_k_—main effect of the k-th level of fatness or muscularity;
(αβ)_ij_—effect of the i-th breed interaction with the j-th parity or insemination order;
(αγ)_ik_—effect of the i-th breed interaction with the k-th level of fatness or muscularity;
(βγ)_jk_—effect of the j-th parity or insemination order interaction with the k-th level of fatness or muscularity;
(αβγ)_ijk_—effect of the i-th breed interaction with the j-th parity or insemination order and the kth level of fatness or muscularity;
ε_ijk_—random error with a normal distribution with an average of zero and a variance σ^2^.

## 3. Results

The results of fatness measurements (UP2, UP4, UPT and UTŚ) carried out by ultrasound method are presented in Table 2. In the first parity, the highest backfat thickness was noted in hybrid gilts (PL × PLW) (*p* ≤ 0.05). It should be emphasized that, after the first farrowing, during the second parity, both hybrid and PLW sows had less backfat thn PL sows (*p* ≤ 0.05). Moreover, statistically significant differences in the thickness of backfat were found for the first and second parity in the case of inter-breed hybrids (PL × PLW) (*p* ≤ 0.05).

Hybrid gilts had a higher backfat thickness during the first parity compared to PLW sows (*p* ≤ 0.05). After the first farrowing, in the second parity, the thickest backfat was found in PL sows (*p* ≤ 0.05). Statistical analysis confirmed the differences in backfat thickness in hybrid females between the first and second parity (*p* ≤ 0.05).

During the first parity, the largest area of fat over Longissimus dorsi muscle was recorded in hybrid gilts (PL × PLW) compared to PL (*p* ≤ 0.05) and PLW (*p* ≤ 0.01). At the moment of the second insemination, the highest results in the range of fat area were noted in PL sows (*p* ≤ 0.01). It should be emphasized that, in the case of the PLW breed and the parameter of fat surface area over LD muscle, statistically significant differences were found between the second and third parity (*p* ≤ 0.05). Statistical differences were also confirmed for this parameter in PL × PLW females between the first and second parity (*p* ≤ 0.05).

The percentage of intramuscular fat in the Longissimus dorsi muscle was calculated using Designer Genes Technologies software compatible with the Aloka SSD-500 ultrasonograph. The analysis of numerical data did not reveal any statistically significant differences, both between the breeds (*p* > 0.05) and related to the parity (*p* > 0.05). The recorded intramuscular fat content was very similar for all studied genetic groups. It should be emphasized, however, that the intramuscular fat content increased gradually with subsequent parities. The limiting level of significance in the case of the greatest differences between the mean values in the case of the UTŚ parameter was *p* = 0.12.

The results of ultrasound measurements of muscularity level (UP4M, USLD, UPLD) are presented in Table 3. In the first parity, the highest depth of LD muscle was observed in hybrid gilts (PL × PLW), however, compared to other breeds it was not statistically significant (*p* > 0.05). The highest values of UP4M parameter were also observed in hybrid sows in the case of second and third parity, but, in this case, the observed differences in relation to PLW and PL sows were statistically confirmed (*p* ≤ 0.05).

Statistical analysis demonstrated a number of differences between subsequent parities in all examined pig breeds. In the case of the PL breed, the statistically confirmed difference in LD muscle depth between the first and third parity was shown (*p* ≤ 0.05). It was observed, in the case of PLW sows, that the depth of the Longissimus dorsi muscle measured in the third parity was significantly higher compared to the results obtained in the first and second parities (*p* ≤ 0.01 and *p* ≤ 0.05, respectively). Moreover, statistically confirmed differences in LD muscle depth were found between the hybrid gilts inseminated for the first time and the hybrid sows inseminated for the third time (*p* ≤ 0.01).

In the case of the USLD parameter, no statistically confirmed inter-breed differences were found (*p* > 0.05). A number of statistically confirmed differences related to the parity were observed. A significant increase in Longissimus dorsi muscle width was observed in each of the breeds studied in subsequent parities.

The highest value of the USLD parameter in the PL breed was recorded in the third parity (*p* ≤ 0.05), and the lowest value in the first one (*p* ≤ 0.05). A similar trend was observed in the analysis of LD muscle width data for the sows of the PLW breed and hybrid females (PL × PLW). In both cases, the highest results in the range of this parameter were recorded at the third farrowing (*p* ≤ 0.01). At the first parity, the width of Longissimus dorsi muscle was the lowest (*p* ≤ 0.01). Moreover, statistically confirmed lower values of the width of Longissimus dorsi muscle were observed for the PLW breed and hybrids (PL × PLW) in the second parity compared to the third one (*p* ≤ 0.05).

It should be noted that the analysis of the UPLD parameter did not show any inter-breed differences (*p* > 0.05). However, differences in the surface area of the Longissimus dorsi muscle were observed between the first and third parity in all experimental breeds. The largest area of LD muscle was recorded at the third parity in hybrid sows (PL × PLW) (*p* ≤ 0.05). The lowest values in the UPLD parameter range, below 60 cm^2^, were recorded in pure-bred PL gilts and PLW gilts inseminated for the first time (*p* ≤ 0.05; *p* ≤ 0.01, respectively).

Table 4 shows the influence of the degree of fatness determined by ultrasound method on reproductive parameters. The relationship between backfat thickness and fat surface over the Longissimus dorsi muscle and reproductive indices was statistically confirmed. Females in which the backfat thickness at UP2 was above 22.25 mm, the backfat thickness at UP4 was above 17.36 mm and the fat area above Longissimus dorsi muscle was above 25.81 cm^2^ at the time of insemination were characterized by the best reproductive parameters. Gilts and sows classified in these groups achieved the highest, at almost 92%, farrowing rate compared to the groups with lower UP2, UP4 and UPT parameters (*p* ≤ 0.05). In addition, the highest number of piglets born in total (UP2 and UP4 *p* ≤ 0.05; UPT *p* ≤ 0.01), the number of alive-born piglets (UP2 and UP4 *p* ≤ 0.05; UPT *p* ≤ 0.01), the lowest number of dead-born piglets (UP2 and UP4 *p* ≤ 0.05; UPT *p* ≤ 0.01), the highest number of weaned piglets (*p* ≤ 0.01) and the lowest mortality of piglets until the weaning day (*p* ≤ 0.05) were observed in these females.

The heaviest litters were produced (*p* ≤ 0.05; *p* ≤ 0.01) and reared (*p* ≤ 0.01) by females which backfat thickness (UP2 and UP4) and fat area over LD muscle (UPT) at the time of insemination were higher than the mean characteristic of the studied population. Piglets from these gilts and sows had a higher body weight at birth (*p* ≤ 0.01). A similar trend was observed in the mean piglets’ weight at weaning (UP2 *p* ≤ 0.05; UP4 and UPT *p* ≤ 0.01) and mean daily gains (UP2 and UPT *p* ≤ 0.05; UP4 *p* ≤ 0.01). It is worth noting that females with above average backfat thickness and fatty tissue surface over Longissimus dorsi muscle had a shorter weaning-to-service interval (*p* ≤ 0.05).

Table 5 presents the influence of the degree of muscularity determined by ultrasound method on the parameters of reproductive performance in the studied population. The analysis of ultrasound measurements of LD muscle performed during insemination showed a significant influence of depth, width and surface of this muscle on reproductive parameters.

Females with the depth of Longissimus dorsi muscle during insemination more than 61.32 mm, width more than 136.08 mm and area more than 63.66 cm^2^, achieved worse reproductive results in the range of farrowing rate (*p* ≤ 0.05), number of piglets born in total (*p* ≤ 0.05; *p* ≤ 01), number of alive-born piglets (*p* ≤ 0.05; *p* ≤ 0.01), number of dead-born piglets (*p* ≤ 0.05), number of weaned piglets (*p* ≤ 0.05; *p* ≤ 0.01) and piglet mortality until the weaning day (*p* ≤ 0.05). It should be noted that gilts and sows in which muscularity was above average gave birth to piglets with low body weight (*p* ≤ 0.05). Moreover, piglets from these females also had significantly lower weight at weaning (*p* ≤ 0.05) and worse body weight gains (*p* ≤ 0.05). The described groups of gilts and sows gave birth to litters of low weight, and statistical analysis confirmed that this was mainly due to the area of LD muscle (*p* ≤ 0.05). In the case of the total litter weight at weaning, the lowest values of this parameter were also recorded in more muscular females (*p* ≤ 0.01). It should be noted that sows with greater muscularity had a longer weaning-to-service interval (*p* ≤ 0.05).

The summarized results of breed effect on reproductive parameters are presented in Table 6.

In general, the best results in the range of all studied indices were obtained by PL × PLW females. The differences were statistically significant compared to PLW breed (*p* ≤ 0.05), except the number of dead-born piglets, piglet mortality, mean piglet weight at weaning as well as parameters related to farrowing and weaning-to-service interval length. The results obtained for PL breed were insignificant compared to two other genotypes, except for mean piglet weight at birth, which differed statistically from hybrid sows.

## 4. Discussion

A very dynamic development of ultrasonography has been observed in recent years, and it is used to examine and image the tissues in human and veterinary medicine, or for the purpose of in vivo measurements of animals in order to characterize their performance [7]. Ultrasonography is a non-invasive method of imaging diagnostics, which allows to obtain an image of the cross-section of the examined object and its advantage is the lack of harmful side effects on the organism of both the researcher and the examined person. The ultrasound technique is very precise, very accurate and allows the detection of even small changes in the examined organs and tissues. Increasingly lower costs of ultrasound equipment and high mobility have made ultrasound scanners more and more popular in pig research [7,15].

One of the devices used for the intravital ultrasound examination of animals is Aloka SSD-500. This device has been widely used in farm animal studies. The device gained particular popularity in research on cattle [16,17,18], as well as on pigs [7,13,19,20]. The spectrum of research conducted with this device was very broad and most often referred to the determination of degree of oocytes development [17], fetus development in different stages [18], and the structure of mammary glands [16]. In addition to its use in the broadly understood diagnostics of reproductive performance, the Aloka SSD-500 is now also used to assess slaughter performance. It is most commonly used to evaluate backfat thickness and Longissimus dorsi muscle width [13,20,21]. Studies with the use of the Aloka ultrasound scanner, aiming to evaluate the degree of fatness and muscularity, allowed even an intravital estimation of the meat content in the carcass [19]. The study conducted by Rempel et al. [22] suggests that, despite the better selection procedures observed currently in animal breeding, there is still a relationship between body condition expressed as fatness or muscularity degree, and reproductive parameters, i.e., litter features. The results of our study in the range of fatness degree are slightly different from that presented by Tyra and Żak [23]. In the study on the domestic pig population, the authors obtained lower results and also noted the highest backfat thickness for the Puławska breed and line 990, 19.20 and 18.30 mm, respectively, and a higher degree of fatness (15.20 mm) was noted for PLW breed compared to PL breed (14.20 mm). However, this was quite the opposite in the case of our study: the difference was almost 1 mm.

In turn, in another study on Polish breeds, Tyra et al. [13] confirmed lower backfat thickness at UP4 compared to UP2 for PL and PLW breeds, which is consistent with the results of our study. The authors showed that the mean backfat thickness measured behind the last rib at the border of thoracic and lumbar vertebrae 8 cm from the middle line of the back was 14.90 mm for PL breed, and 14.50 mm for PLW. The lower values of this parameter obtained by the authors may be associated with lower body weight of the examined animals.

Ultrasonographic examination of fat area under LD muscle was performed by Tyra et al. [20], and the results were below 20.00 cm^2^, which was related to the low weight of the examined animals, i.e., 100 kg. The authors demonstrated strong correlation between this parameter and backfat thickness at P2 and P4 measured during carcass dissection. Correlation coefficients were at a similar level for these two analyzed parameters and amounted to about 0.53. Moreover, the authors noted that an increase in LD muscle depth is accompanied by a significant decrease in the fat area above the muscle. Subsequent study of UPT parameter conducted by Tyra et al. [20] in the domestic population using the Aloka SSD-500 apparatus showed inter-breed differences. The authors noted the highest values for Duroc breed (21.3 cm^2^). The lowest fat area over the Longissimus dorsi was recorded for Pietrain breed (14.1 cm^2^). Maternal breeds, i.e., Polish Landrace and Polish Large White, were characterized by intermediate results for this trait. However, PL breed had a slightly higher fatness degree compared to PLW. The authors calculated that PL gilts had, on average, 20.6 cm^2^ of fat area over Longissimus dorsi muscle, while this value for PLW gilts was about 19.4 cm^2^.

Intramuscular fat in our study was determined in a similar manner as in the study of Bahelka et al. [24]. The results of these authors confirm that Aloka SSD-500 device with 3.5 MHz UST-5044 head, with a signal amplification of 80%, allows to determine intramuscular fat content with a very high accuracy. Intramuscular fat content determined by means of ultrasound amounted to 2.28%, whereas the result obtained based on chemical analysis was 2.22%.

The results of intramuscular fat measurements obtained in our study are similar to the results obtained by Orzechowska et al. [25], who showed that in the PL breed it was 1.72%, and 1.71% in PLW breed. Moreover, the study confirmed that this parameter value changes with an increase in animals’ muscularity. In the case of PL breed, the animals with the highest leanness, above 60%, were characterized by the lowest intramuscular fat level (1.71%). A similar tendency was also observed in PL breed, where the carcasses of the most muscular pigs were rated the highest (class S), and had an intramuscular fat level of 1.68%.

Slightly different results of intramuscular fat measurements were obtained by Tyra and Żak [23], who observed higher intramuscular fat content in Longissimus dorsi muscle in PLW breed (1.84%) compared to PL (1.76%). In our study, PLW gilts and sows also had a slightly higher intramuscular fat content in LD muscle. Moreover, the authors recorded very high results in this parameter in Duroc (2.23%) and Puławska (2.17%) breeds. Jankowiak et al. [26] studied the relationship between carcass fatness and intramuscular fat content and fatty acid profile in pig meat. The study was conducted on Puławska breed and hybrids from PLW and PL crossbreeding. Intramuscular fat content in Puławska breed was high and amounted to 1.87%, while in hybrids a lower result was found (1.72%).

With regards to the muscularity degree examined in this study, similar results in the scope of LD muscle depth were obtained by Knecht et al. [27]. The authors demonstrated that hybrid gilts were characterized by an LD muscle depth of 61.47 mm on average. In the case of our study, the average depth of Longissimus dorsi muscle in gilts from such cross-breeding was about 60 mm. This difference can be explained by higher representativeness of the research group, due to more numerous groups of animals participating in the experiment.

It is worth emphasizing that no inter-breed differences in the case of the UPLD parameter were demonstrated in our study. Orzechowska et al. [25] observed that PL breed is characterized by a larger area of the tenderloin eye compared to PLW breed. LD muscle area in PL breed was 56.50 cm^2^, while in PLW it was 55.2 cm^2^. In our study, PL sows, which were inseminated for the second and third time, also had a larger area of the tenderloin eye compared to PLW sows. Moreover, the results of the study of the abovementioned authors showed that with leanness exceeding 60%; this parameter was 56.70 cm^2^ for PL breed, and 57.40 cm^2^ for PLW. The study conducted by Tyra and Żak [23] also showed that PL breed is characterized by a larger surface area of tenderloin eye compared to PLW breed. According to these authors, the area of LD muscle in PL was 53.40 cm^2^, whereas in PLW it was 52.70 cm^2^. The results obtained are lower than those presented in Table 3 because the animals had a lower body weight (about 100 kg).

Tyra et al. [13] examined the differences in the results of LD muscle surface measurements using ultrasound and dissection methods. In the case of the PL breed, the ultrasound measurement of tenderloin eye area gave the result of 49.9 cm^2^, while the dissection measurement result was 52.2 cm^2^. The examination of LD muscle area in PLW breed in the case of the ultrasound method gave the result of 51.10 cm^2^, whereas after dissection the result was 52.70 cm^2^. The differences were therefore insignificant and related to the measurement methodology. Krška et al. [19] also studied tenderloin eye area using the ultrasound method with Aloka SSD-500 apparatus. The authors determined meat content in the carcass on the basis of intravital measurements performed with Piglog-105 probe, SonoMark SM-100 scanner and Aloka SSD-500 apparatus. The highest accuracy was found in the case of Aloka SSD-500, with the leanness estimated using this method amounting to 55.83%, while value determined by dissection was 55.67%.

The results obtained show that an increase in the degree of fatness during insemination may determine the improvement in reproductive indices. De Rensis et al. [28] observed in their study no relationship between backfat thickness and farrowing rate, which is contrary to our results. Matysiak et al. [9] stated that the number of alive-born piglets and the number of piglets at weaning are positively correlated with the backfat thickness on a level of 0.31 and 0.24, which means that females with thicker backfat, similarly to our study, gave birth to more piglets and recorded a higher number of piglets at weaning. Holm et al. [4] point out that when energy reserves are too low (the female has a lower degree of fatness during the first insemination), the development and implantation of embryos in the uterus may not proceed properly, which increases the number of embryos that are resorbed in the uterus and, as a result, contributes to a reduction in the number of alive-born piglets. Bergsma et al. [29] claim that lactation can be a period with maximum energy expenditure, and this may limit reproductive performance, e.g., litter growth. Vanroose et al. [30] and Knecht et al. [6] also pay attention to other factors influencing embryo resorption, such as viral infections, stress, poor nutrition or seasonality of reproduction.

In our study, it was found that the lowest birth weights of piglets were recorded in gilts and sows, which were characterized by lower or equal to the mean population thickness of backfat and the surface of fat tissue over LD muscle during insemination (UP2 ≤ 22.25 mm, UP4 ≤ 17.36 mm, UPT ≤ 25.81 cm). It should be emphasized that a lower degree of gilt fatness during insemination may interfere with fetal development and worsen fetal growth, and may also affect the lower weight of piglets at birth, which probably contributed to the poorer reproductive performance indices in this range. Milligan et al. [31] emphasize that a low body weight at birth is accompanied by an increase in the number of dead-born piglets and an increase in mortality during the rearing period, which is associated with a very high physiological effort of the gilt organism.

Immediately after parturition, reserves of energy accumulated earlier are released in the form of adipose tissue. The metabolism of gilts and sows, oriented for a long period of pregnancy to store energy reserves, has problems with switching in a very short period of time to the use of nutrients taken from feed for milk production. Weaker gestational anabolism is likely to result in weaker lactation, which may result in a reduction in the number of weaned piglets, inter alia due to insufficient milk production, piglets may also have a lower weaning weight due to insufficient feed intake [32]. It was also demonstrated in the study by Strathe et al. [32] that backfat thickness and backfat losses during lactation did not affect the number of piglets born in subsequent parity, which is contrary to our findings, however, the differences could be attributed to different rearing strategies, breed or environmental conditions.

The study by Tummaruk et al. [3] confirm that gilts with a high growth rate have more numerous and heavier litters than sows. The authors suggest that this may be related to higher feed intake resulting in a thicker backfat. The gilts with a higher growth rate consume more feed during their growth, thus their health and nutritional status is better, which is then reflected in improved reproductive parameters compared to gilts with a lower growth rate. Moreover, Holm et al. [4] state that the thickness of backfat considered as a source of energy for sows may also play an important role in subsequent breeding cycles. Szulc et al. [33] report that too high a level of female fatness during mating/insemination may lead to hormonal disorders. In the case of these authors’ study, it was noted that females with the thickest backfat (>15 mm) gave birth to the lowest number of alive piglets. The authors explained their results by the characteristic transformation of estradiol to estriol, a weaker estrogen that occurs in more fattened females. The opposite effect was observed in our study. Females in which backfat thickness was higher than the average of population at both examined points produced and reared the highest number of piglets characterized by very high weights at birth and weaning. Tummaruk et al. [3] obtained similar results in their study. However, the differences can be due to genetics. It may be supposed that, in the case of a very lean genetics, the benefit of a certain threshold of backfat is positive, like in the current study, but excess backfat might indicate that the animal is too heavy, too old or in heat, and that might be the explanation for the different findings.

Selection aimed at improving fattening and slaughter traits led to a significant increase in the muscularity of the animals, but at the expense of a reduction in fatness level, which led to a deterioration in reproductive capacity. Many authors confirmed a very strong positive correlation between backfat thickness and litter size, e.g., [3,34,35,36]. Our study confirms the thesis that females with higher backfat thickness at insemination may produce more piglets with higher birth weight. A similar trend can also be observed in the analysis of fat surface over LD muscle. This can be explained by the fact that the beneficial effect of LD muscle could be a direct effect of weight. That higher body weight increases the size of reproductive organs as well as LD muscle.

Kawęcka et al. [37] found negative correlations between gilts’ muscularity and their subsequent reproductive performance in three consecutive litters, which means that too much muscularity caused a deterioration in reproductive indices. A similar trend was observed in our study. Decreasing the muscularity level in the range of UP4M, USLD and UPLD parameters resulted in an improvement in reproductive performance indices. It should be also born in mind that fat reserves can be mobilized relatively easily, while muscle store mobilization for energy purposes is quite the opposite [38].

## 5. Conclusions

The measurements using Aloka SSD-500 demonstrated the highest fat thickness, as well as the highest fat area over LD muscle in hybrid gilts (PL × PLW) in the first parity. The muscularity of pure-bred gilts was lower compared to hybrid gilts. Fatness degree influenced the reproductive parameters of females in which the backfat thickness in UP2 was above 22.25 mm, in UP4 point above 17.36 mm and the fat area over LD muscle was above 25.81 cm^2^. These females achieved a better farrowing rate and higher numbers of born piglets. Decreased mortality, higher gains of piglets and higher body weight at weaning were observed, and the weaning to service interval was shortened. Intramuscular fat content did not affect reproductive parameters. Muscularity evaluated by ultrasound affected reproductive performance parameters, except gestation period. Females with too high muscularity were characterized by the lowest reproductive indices.

## Figures and Tables

**Table 1:** Linear, surface and quality measurements taken with Aloka SSD-500 ultrasound system.

Measurement	Description of the Measurement Point
UP2	Backfat thickness at point P2—backfat thickness at the last rib, at the junction of the thoracic and lumbar vertebrae—3 cm from the midline of the spine (mm).
UP4	Backfat thickness at point P4—backfat thickness at the last rib, at the junction of the thoracic and lumbar vertebrae—8 cm from the midline of the spine (mm).
UPT	Fat cross-sectional area on the surface of the Longissimus dorsi muscle (cm^2^).
UTŚ	Intramuscular fat content in the Longissimus dorsi muscle (%).
UP4M	Depth of Longissimus dorsi muscle at point P4—height of eye of the loin, at the junction of the thoracic and lumbar vertebrae—8 cm from the midline of the spine (mm).
USLD	Width of the Longissimus dorsi muscle (mm).
UPLD	Cross-sectional area of the Longissimus dorsi muscle (cm^2^).

**Table 2:** Results of fatness measurements carried out using Aloka SSD-500 (breed and parity).

**Parameter UP2**						
**Parity**	**Breed**					
	**Polish Landrace (PL)**		**Polish Large White (PLW)**		**PL × PLW**	
	$\bar{x}$ **(mm)**	**SD**	$\bar{x}$ **(mm)**	**SD**	$\bar{x}$ **(mm)**	**SD**
1	22.26	±4.70	21.17 ^y^	±3.99	23.08 ^a,x^	±3.89
2	23.29 ^x^	±4.02	21.19 ^y^	±4.16	21.18 ^b,y^	±4.79
3	22.24	±4.76	22.93	±4.52	22.94	±4.43
**Parameter UP4**						
**Parity**	**Breed**					
	**PL**		**PLW**		**PL × PLW**	
	$\bar{x}$ **(mm)**	**SD**	$\bar{x}$ **(mm)**	**SD**	$\bar{x}$ **(mm)**	**SD**
1	17.54	±3.60	16.35 ^y^	±4.21	18.56 ^a,x^	±3.44
2	18.98 ^x^	±3.34	15.67 ^y^	±4.03	16.26 ^b,y^	±4.15
3	17.65	±3.65	17.07	±3.89	18.12	±3.38
**Parameter UPT**						
**Parity**	**Breed**					
	**PL**		**PLW**		**PL × PLW**	
	$\bar{x}$ **(cm^2^)**	**SD**	$\bar{x}$ **(cm^2^)**	**SD**	$\bar{x}$ **(cm^2^)**	**SD**
1	25.19 ^y^	±6.61	23.34 ^Y^	±6.85	28.20 ^a,X,x^	±7.43
2	27.23 ^X^	±7.45	23.02 ^b,Y^	±6.45	25.10 ^b^	±7.11
3	26.46	±8.96	25.96 ^a^	±7.66	27.83	±6.78
**Parameter UTŚ**						
**Parity**	**Breed**					
	**PL**		**PLW**		**PL × PLW**	
	$\bar{x}$ **(%)**	**SD**	$\bar{x}$ **(%)**	**SD**	$\bar{x}$ **(%)**	**SD**
1	1.67	±0.66	1.68	±0.60	1.71	±0.67
2	1.71	±0.78	1.73	±0.74	1.75	±0.72
3	1.77	±0.83	1.79	±0.80	1.82	±0.87

$\bar{x}$—mean value. SD—standard deviation. a,b—in the same column, denote statistically significant differences between parity for a given breed, with *p* ≤ 0.05. x,y—in the same line, denote statistically significant differences between breeds, for a given parity, with *p* ≤ 0.05. X,Y—in the same line, denote statistically significant differences between breeds, for a given parity, with *p* ≤ 0.01.

**Table 3:** Results of muscularity measurements carried out using Aloka SSD-500 (breed and parity).

**Parameter UP4M**						
**Parity**	**Breed**					
	**PL**		**PLW**		**PL × PLW**	
	$\bar{x}$ **(mm)**	**SD**	$\bar{x}$ **(mm)**	**SD**	$\bar{x}$ **(mm)**	**SD**
1	57.85 ^b^	±6.16	57.73 ^B^	±6.04	59.94 ^B^	±6.75
2	59.52	±6.52	59.42 ^b,y^	±7.99	63.47 ^x^	±5.83
3	62.59 ^a,y^	±6.62	64.53 ^A,a^	±5.62	66.80 ^A,x^	±4.23
**Parameter USLD**						
**Parity**	**Breed**					
	**PL**		**PLW**		**PL × PLW**	
	$\bar{x}$ **(mm)**	**SD**	$\bar{x}$ **(mm)**	**SD**	$\bar{x}$ **(mm)**	**SD**
1	131.68 ^b^	±9.01	131.83 ^B^	±7.06	132.86 ^B^	±8.97
2	135.10	±8.26	134.32 ^b^	±7.19	135.38 ^b^	±8.52
3	139.40 ^a^	±8.32	141.35 ^A,a^	±7.84	142.76 ^A,a^	±9.24
**Parameter UPLD**						
**Parity**	**Breed**					
	**PL**		**PLW**		**PL × PLW**	
	$\bar{x}$ **(cm^2^)**	**SD**	$\bar{x}$ **(cm^2^)**	**SD**	$\bar{x}$ **(cm^2^)**	**SD**
1	59.37 ^b^	±9.16	58.90 ^B^	±8.92	62.51 ^b^	±8.82
2	61.51	±10.01	61.59	±9.38	65.16	±9.93
3	66.58 ^a^	±10.49	67.70 ^A^	±11.40	69.58 ^a^	±10.26

$\bar{x}$—mean value. SD—standard deviation. a,b—in the same column, denote statistically significant differences between parity for a given breed, with *p* ≤ 0.05; A,B—in the same column, denote statistically significant differences between parity for a given breed, with *p* ≤ 0.01; x,y—in the same line, denote statistically significant differences between breeds, for a given parity, with *p* ≤ 0.05.

**Table 4:** An effect of fatness degree determined by ultrasound method on reproductive parameters of sows.

$\mathrm{Reproduction}\;\mathrm{Parameter}\;(\bar{x},s)$	Degree of Fatness Determined by Aloka SSD-500 Apparatus							
	UP2		UP4		UPT		UTŚ	
	≤22.25 mm	>22.25 mm	≤17.36 mm	>17.36 mm	≤25.81 cm^2^	>25.81 cm^2^	≤1.74%	>1.74%
Farrowing rate (%)	87.89 ^b^	91.90 ^a^	87.99 ^b^	91.99 ^a^	87.50 ^b^	91.56 ^a^	88.00	90.30
Number of piglets born in total (head)	10.34 ^b^ ± 0.83	11.14 ^a^ ± 0.91	10.30 ^b^ ± 0.85	11.25 ^a^ ± 0.92	10.20 ^B^ ± 0.87	11.30 ^A^ ± 0.94	10.45 ± 0.88	10.99 ± 0.95
Number of alive-born piglets (head)	10.04 ^b^ ± 0.85	10.94 ^a^ ± 0.77	9.97 ^b^ ± 0.79	11.06 ^a^ ± 0.76	9.82 ^B^ ± 0.91	11.08 ^A^ ± 0.74	10.19 ± 0.70	10.74 ± 0.82
Number of dead-born piglets (head)	0.30 ^b^ ± 0.41	0.20 ^a^ ± 0.39	0.33 ^b^ ± 0.35	0.19 ^a^ ± 0.43	0.38 ^B^ ± 0.40	0.22 ^A^ ± 0.38	0.26 ± 0.41	0.25 ± 0.36
Number of weaned piglets (head)	9.58 ^B^ ± 0.77	10.59 ^A^ ± 0.69	9.56 ^B^ ± 0.65	10.76 ^A^ ± 0.68	9.39 ^B^ ± 0.75	10.77 ^A^ ± 0.67	9.75 ± 0.60	10.33 ± 0.63
Mortality of piglets until weaning day (head)	0.46 ^b^ ± 0.53	0.35 ^a^ ± 0.51	0.41 ^b^ ± 0.45	0.30 ^a^ ± 0.48	0.43 ^b^ ± 0.54	0.31 ^a^ ± 0.47	0.44 ± 0.50	0.41 ± 0.43
Total litter weight at 1st day (kg)	14.56 ^b^ ± 2.44	17.50 ^a^ ± 2.13	14.26 ^b^ ± 2.32	17.47 ^a^ ± 2.20	14.14 ^B^ ± 2.47	17.95 ^A^ ± 2.05	14.98 ± 2.19	16.11 ± 2.28
Average piglet weight at 1st day (kg)	1.45 ^B^ ± 0.25	1.60 ^A^ ± 0.20	1.43 ^B^ ± 0.27	1.58 ^A^ ± 0.18	1.44 ^B^ ± 0.23	1.62 ^A^ ± 0.20	1.47 ± 0.25	1.50 ± 0.29
Total litter weight at weaning (kg)	80.57 ^B^ ± 10.90	91.07 ^A^ ± 9.85	80.30 ^B^ ± 10.29	92.86 ^A^ ± 9.81	78.78 ^B^ ± 10.83	92.84 ^A^ ± 9.89	82.39 ± 10.50	87.70 ± 9.51
Average piglet weight at weaning (kg)	8.41 ^b^ ± 0.44	8.60 ^a^ ± 0.48	8.40 ^B^ ± 0.51	8.63 ^A^ ± 0.53	8.39 ^B^ ± 0.52	8.62 ^A^ ± 0.51	8.45 ± 0.47	8.49 ± 0.50
Average body gains of piglets (g)	248.57 ^b^ ± 9.85	250.00 ^a^ ± 9.59	248.93 ^B^ ± 10.36	251.79 ^A^ ± 9.55	248.21 ^b^ ± 9.86	250.00 ^a^ ± 9.66	249.29 ± 10.47	249.64 ± 9.57
Gestation period (days)	114.62 ± 2.25	114.09 ± 2.70	114.55 ± 2.44	114.26 ± 2.39	114.72 ± 2.51	114.10 ± 2.73	114.65 ± 2.44	114.60 ± 2.71
Weaning to service interval (days)	11.12 ^b^ ± 10.33	9.77 ^a^ ± 9.78	11.00 ^b^ ± 11.02	9.65 ^a^ ± 9.99	11.20 ^b^ ± 10.31	9.84 ^a^ ± 9.90	10.60 ± 10.45	10.29 ± 10.25

a, b—in the same line denote statistically significant differences between the degrees of fatness, with *p* ≤ 0.05, A, B—in the same line denote statistically significant differences between the degrees of fatness, with *p* ≤ 0.01.

**Table 5:** An effect of muscularity degree determined by ultrasound method on the reproductive parameters of sows.

$\mathrm{Reproduction}\;\mathrm{Parameter}\;(\bar{x},s)$	Degree of Muscularity Determined by Aloka SSD-500 Apparatus					
	UP4M		USLD		UPLD	
	≤61.32 mm	>61.32 mm	≤136.08 mm	>136.08 mm	≤63.66 cm^2^	>63.66 cm^2^
Farrowing rate (%)	92.59 ^a^	88.58 ^b^	91.29 ^a^	87.11 ^b^	92.79 ^a^	88.78 ^b^
Number of piglets born in total (head)	10.99 ^a^ ± 0.89	10.15 ^b^ ± 0.85	11.02 ^A^ ± 0.90	9.97 ^B^ ± 0.89	11.14 ^A^ ± 0.91	10.05 ^B^ ± 0.88
Number of alive-born piglets (head)	10.81 ^a^ ± 0.75	9.87 ^b^ ± 0.79	10.82 ^A^ ± 0.71	9.67 ^B^ ± 0.69	10.93 ^A^ ± 0.70	9.73 ^B^ ± 0.78
Number of dead-born piglets (head)	0.18 ^a^ ± 0.34	0.28 ^b^ ± 0.31	0.20 ^a^ ± 0.39	0.30 ^b^ ± 0.42	0.21 ^a^ ± 0.39	0.32 ^b^ ± 0.47
Number of weaned piglets (head)	10.55 ^a^ ± 0.65	9.50 ^b^ ± 0.61	10.52 ^A^ ± 0.65	9.26 ^B^ ± 0.72	10.65 ^A^ ± 0.61	9.34 ^B^ ± 0.67
Mortality of piglets until weaning day (head)	0.26 ^a^ ± 0.41	0.37 ^b^ ± 0.45	0.30 ^a^ ± 0.44	0.41 ^b^ ± 0.47	0.28 ^a^ ± 0.40	0.39 ^b^ ± 0.50
Total litter weight at 1st day (kg)	16.76 ± 2.22	14.21 ± 2.25	16.66 ± 2.09	13.83 ± 2.55	17.27 ^a^ ± 2.20	14.30 ^b^ ± 2.24
Average piglet weight at 1st day (kg)	1.55 ^a^ ± 0.23	1.44 ^b^ ± 0.27	1.54 ^a^ ± 0.19	1.43 ^b^ ± 0.29	1.58 ^a^ ± 0.26	1.47 ^b^ ± 0.30
Total litter weight at weaning (kg)	90.31 ^A^ ± 10.24	79.80 ^B^ ± 10.29	90.37 ^A^ ± 9.80	77.97 ^B^ ± 10.99	91.38 ^A^ ± 9.78	78.55 ^B^ ± 10.28
Average piglet weight at weaning (kg)	8.56 ^a^ ± 0.44	8.40 ^b^ ± 0.52	8.59 ^a^ ± 0.55	8.42 ^b^ ± 0.45	8.58 ^a^ ± 0.53	8.41 ^b^ ± 0.47
Average body gains of piglets (g)	250.36 ^a^ ± 10.35	248.57 ^b^ ± 10.39	251.79 ^a^ ± 9.78	249.64 ^b^ ± 9.90	250.00 ^a^ ± 9.87	247.86 ^b^ ± 10.67
Gestation period (days)	114.20 ± 2.45	114.60 ± 2.57	114.15 ± 2.80	114.71 ± 2.75	114.03 ± 2.77	114.76 ± 2.50
Weaning to service interval (days)	9.81 ^a^ ± 9.94	11.16 ^b^ ± 11.32	10.00 ^a^ ± 9.95	11.36 ^b^ ± 9.97	9.93 ^a^ ± 10.32	11.28 ^b^ ± 10.89

a, b—in the same line denote statistically significant differences between the degrees of muscularity, with *p* ≤ 0.05, A, B—in the same line denote statistically significant differences between the degrees of muscularity, with *p* ≤ 0.01.

**Table 6:** Breed effect on reproduction parameters.

$\mathrm{Reproduction}\;\mathrm{Parameter}\;(\bar{x},s)$	Breed		
	PL	PLW	PL × PLW
Farrowing rate (%)	90.22	88.70 ^b^	91.30 ^a^
Number of piglets born in total (head)	10.20 ± 0.77	9.90 ^b^ ± 0.81	11.00 ^a^ ± 0.72
Number of alive-born piglets (head)	10.00 ± 0.70	9.60 ^b^ ± 0.80	10.80 ^a^ ± 0.75
Number of dead-born piglets (head)	0.20 ± 0.31	0.25 ± 0.37	0.15 ± 0.31
Number of weaned piglets (head)	9.70 ± 0.50	9.55 ^b^ ± 0.70	10.51 ^a^ ± 0.61
Mortality of piglets until weaning day (head)	0.44 ± 0.44	0.32 ± 0.50	0.38 ± 0.40
Total litter weight at 1st day (kg)	14.99 ± 2.30	13.80 ^b^ ± 2.51	16.95 ^a^ ± 2.09
Average piglet weight at 1st day (kg)	1.43 ^b^ ± 0.27	1.41 ^b^ ± 0.29	1.55 ^a^ ± 0.20
Total litter weight at weaning (kg)	83.20 ± 10.15	78.77 ^b^ ± 10.66	89.40 ^a^ ± 9.99
Average piglet weight at weaning (kg)	8.45 ± 0.39	8.30 ± 0.42	8.59 ± 0.49
Average body gains of piglets (g)	249.00 ± 10.50	248.01 ^b^ ± 9.71	250.19 ^a^ ± 9.89
Gestation period (days)	114.76 ± 2.35	114.53 ± 2.19	114.79 ± 2.71
Weaning to service interval (days)	10.70 ± 9.71	10.90 ± 10.01	10.66 ± 9.80

a, b—in the same line denote statistically significant differences between the breeds, with *p* ≤ 0.05.

## References

[1] POLSUS (2018). Results of the Evaluation of Pigs in 2017.

[2] Rozeboom D.W., Pettigrew J.E., Mosel R.L., Cornelius S.G., El Kandelgy S.M. (1996). Influence of gilt age and body composition at first breeding on sow reproductive performance and longevity. J. Anim. Sci..

[3] Tummaruk P., Lundeheim N., Einarsson S., Dalin A.M. (2001). Effect of birth litter size, birth parity number, growth rate, backfat thickness and age at first mating of gilts on their reproductive performance as sow. Anim. Reprod. Sci..

[4] Holm B., Bakken M., Klemetsdal G., Vangen O. (2004). Genetic correlations between reproduction and production traits in swine. J. Anim. Sci..

[5] Kim J.S., Yang X., Pangeni D., Baidoo S.K. (2015). Relationship between backfat thickness of sows Turing late gestation and reproductive efficiency at different parities. Acta Agric. Scand. Sect. A.

[6] Knecht D., Duziński K. (2014). The effect of parity and date of service on the reproductive performance of polish large white × polish landrace (plw × pl) crossbred sows. Ann. Anim. Sci..

[7] Knecht D., Środoń S., Jankowska-Mąkosa A., Duziński K. (2017). Appropriate level of gilt fatness and muscularity during insemination can improve the efficiency of piglet production. Anim. Reprod..

[8] Quinton V.M., Wilton J.W., Robinson J.A., Mathur P.K. (2006). Economic weights for sow productivity traits in nucleus pig populations. Livest. Sci..

[9] Matysiak B., Kawęcka M., Jacynio E., Kołodziej-Skalska A., Pietruszka A. (2010). Relationships between test of gilts before day at first mating on their reproduction performance. Acta Sci. Pol. Zoot..

[10] Grela E., Skomiał J. (2014). Nutritional Recommendations and Nutritional Value of Feedingstuffs for Pigs. Standards of pigs Nutrition.

[B11-animals-10-00794] 11.Journal of Laws of 2010, no 116, item 778, Ordinance of the Minister of Agriculture and Rural Development of 28 June 2010 on minimum conditions for maintaining farm animal species other than those for which the standards of protection are laid down in the European Union regulations.

[B12-animals-10-00794] 12.Journal of Laws of 26 February 2015, item 266, Act of 15 January 2015 on the protection of animals used for scientific and educational purposes.

[13] Tyra M., Szyndler-Nędza M., Eckert R. (2011). Possibilities of using ultrasonography in breeding work with pigs, Part I—Analysis of ultrasonic, ultrasonographic and dissection measurements of the most numerous breeds of pigs raised in Poland. Annals Anim. Sci..

[14] Schulte K.J., Baas T.J., Wilson D.E. (2011). An evaluation of equipment and procedures for the prediction of intramuscular fat in live swine. Anim. Ind. Rep..

[15] Mörlein D., Rosner F., Brand S., Jenderka K.V., Wicke M. (2005). Non-destructive estimation of the intramuscular fat content of the longissimus muscle of pigs by means of spectral analysis of ultrasound echo signals. Meat Sci..

[16] Strzetelski J., Bilik K., Niwińska B., Skrzyński G., Łuczyńska E. (2004). Ultrasound evaluation of the mammary gland structure in preparturient heifers vs performance of first calves. J. Anim. Feed Sci..

[17] Adamiak S.J., Mackie K., Watt R.G., Webb R., Sinclair K.D. (2005). Impact of nutrition on oocyte quality: Cumulative effects of body composition and diet leading to hyperinsulinemia in cattle. Biol. Reprod..

[18] McNeill R.E., Diskin M.G., Sreenan J.M., Morris D.G. (2006). Associations between milk progesterone concentration on different days and with embryo survival during the early luteal phase in dairy cows. Theriogenology.

[19] Krška P., Bahelka I., Demo P., Peškovičová D. (2002). Meat content in pigs estimated by various methods and compared with objective lean meat content. Czech J. Anim. Sci..

[20] Tyra M., Orzechowska B., Żak G. (2005). Relationships between ultrasonic and dissection measurements of backfat thickness and m. Longissimus dorsi of pigs using Piglog 105 and Aloka SSD 500. Ann. Anim. Sci..

[21] Kearns C.F., McKeever K.H., Roegner V., Brady S.M., Malinowski K. (2006). Adiponectin and leptin are related to fat mass in horses. Vet. J..

[22] Rempel L.A., Vallet J.L., Lents C.A., Nonneman D.J. (2015). Measurements of body composition during late gestation and lactation in first and second parity sows and its relationship to piglet production and post-weaning reproductive performance. Livest. Sci..

[23] Tyra M., Żak G. (2010). Characteristics of the Polish breeding population of pigs in terms of intramuscular fat (IMF) content of m. Longissimus dorsi. Ann. Anim. Sci..

[24] Bahelka I., Oravcová M., Peškovičová D., Tomka J., Hanusová E., Lahučký R., Demo P. (2009). Comparison of accuracy of intramuscular fat prediction in live pigs using five different ultrasound intensity levels. Animal.

[25] Orzechowska B., Tyra M., Mucha A., Żak G. (2012). Quality of carcasses from polish large white and polish landrace pigs with particular consideration of intramuscular fat (IMF) content depending on carcass meat percentage. Roczniki Nauk. Zoot..

[26] Jankowiak H., Bocian M., Kapelanski W., Roslewska A. (2010). The relationship between carcass fatness and intramuscular fat content, and fatty acids profile in pig meat. Food Sci. Technol. Qual..

[27] Knecht D., Środoń S., Duziński K. (2014). In vivo evaluation of the fat content and muscularity of gilts with different genotypes using Aloka SSD-500 ultrasound scanner in relation to selected reproductive performance indicators. Roczn. Nauk. Pol. Tow. Zoot..

[28] De Rensis F., Gherpelli M., Superchi P., Kirkwood R.N. (2005). Relationships between backfat depth and plasma leptin during lactation and sow reproductive performance after weaning. Anim. Reprod. Sci..

[29] Bergsma R., Kanis E., Verstegen M.W.A., van der Peet-Schwering C.M.C., Knol E.F. (2009). Lactation efficiency as a result of body composition dynamics and feed intake in sows. Livest. Sci..

[30] Vanroose G., de Kruif A., Van Soom A. (2000). Embryonic mortality and embryo–pathogen interactions. Anim. Reprod. Sci..

[31] Milligan B.N., Dewey C.E., de Grau A.F. (2002). Neonatal-piglet weight variation and its relation to pre-weaning mortality and weight gain on commercial farms. Prevent. Vet. Med..

[32] Strathe A.V., Bruun T.S., Hansen C.F. (2017). Sows with high milk production had both a high feed intake and high body mobilization. Animal.

[33] Szulc K., Knecht D., Jankowska-Mąkosa A., Skrzypczak E., Nowaczewski S. (2013). The influence of fattening and slaughter traits on reproduction in Polish Large White sows. Ital. J. Anim. Sci..

[34] Karsten S., Rohe R., Schulze V., Looft H., Kalm E. (2000). Genetic association between individual feed intake during performance test and reproductions traits in pigs. Archiv Tierz..

[35] Bečková R., Daněk P., Václavková E., Rozkot M. (2005). Influence of growth rate, backfat thickness and meatiness on reproduction efficiency in Landrace gilts. Czech J. Anim. Sci..

[36] Stalder K.J., Saxton A.M., Conatser G.E., Serenius T.V. (2005). Effect of growth and compositional traits on first parity and lifetime reproductive performance in U.S. Landrace sows. Livest. Prod. Sci..

[37] Kawęcka M., Matysiak B., Kamyczek M., Delikator B. (2009). Relationships between growth, fatness and meatiness traits in gilts and their subsequent reproductive performance. Ann. Anim. Sci..

[38] Lewis C.R., Bunter K.L. (2011). Body development in sows, feed intake and maternal capacity. Part2: Gilt body condition before and after lactation, reproductive performance and correlations with lactation feed intake. Animal.

